# GSK-3β and ERK1/2 incongruously act in tau hyperphosphorylation in SPS-induced PTSD rats

**DOI:** 10.18632/aging.102303

**Published:** 2019-09-23

**Authors:** Zhen Wei, Yacoubou Abdoul Razak Mahaman, Feiqi Zhu, Mengjuan Wu, Yiyuan Xia, Kuan Zeng, Ying Yang, Rong Liu, Jian-Zhi Wang, Xiji Shu, Xiaochuan Wang

**Affiliations:** 1 Department of Pathophysiology, School of Basic Medicine, Key Laboratory of Education Ministry of China for Neurological Disorders, Tongji Medical College, Huazhong University of Science and Technology, Wuhan 430030, China; 2 Cognitive Impairment Ward of Neurology Department, The Third Affiliated Hospital of Shenzhen University, Shenzhen 518001, Guangdong Province, China; 3 Department of Pathology and Pathophysiology, School of Medicine, Jianghan University, Wuhan 430056, China; 4 Co-innovation Center of Neuroregeneration, Nantong University, Nantong, JS 226001, China

**Keywords:** post-traumatic stress disorder (PTSD), single prolonged stress (SPS), Alzheimer disease (AD), tau phosphorylation, GSK-3β

## Abstract

Post-traumatic stress disorder (PTSD) manifests in neurocognitive deficits in association with increased tau deposition, which mainly consist of phosphorylated tau in Alzheimer disease (AD) brain. However, the exact mechanism of PTSD inducing tau hyperphosphorylation remains unclear and therefore no effective treatment options are currently available. We here show that employing single prolonged stress (SPS), as a consensus PTSD model, induced a typical anxiety and abnormal hyperphosphorylation of tau at Ser202/Thr205 (AT8) and Ser404 but not at Ser199 and Ser396 in the hippocampus compared to the control rats. Furthermore, there was a decrease in the level of inactivated phosphorylated GSK-3β at Ser9, an increase in the level of activated phosphorylated GSK-3β at Thr216 and an obvious decrease in the level of activated phosphorylated ERK1/2, but no alterations in CaMKII and PP2A in hippocampus of SPS rats. On the other hand, the levels of both phosphorylated AKT and total SGK1, stress- and GSK-3β/ERK1/2-related proteins, were down-regulated. Interestingly, Overexpression of SGK1 increased the level of phosphorylated ERK1/2 and led to tau hyperphosphorylation at Ser199 and Ser396. These findings suggest that SPS exposure results in differential tau phosphorylation at different sites probably due to incongruous action between AKT-related GSK-3β activation and SGK1-related ERK1/2 inactivation, suggesting a link between SPS-induced PTSD and AD-associated tau pathogenic mechanisms.

## INTRODUCTION

Posttraumatic stress disorder (PTSD) is a mental disorder that can develop after a person is exposed to a traumatic event including mental and physical assaults, and it severely impairs the quality of life of the affected individual. According to the fifth version of the diagnostic and statistical manual of mental disorders (DSM - V), PTSD can be diagnosed with some typical symptoms for over one month [1] as follows: flashbacks, intrusive thoughts, avoiding trauma-like scenes or people, negative alterations in mood and insomnia with nightmares, which are related with stress-induced changes or an inadequate adaptation of neurobiological systems. PTSD patients show chronic stress responses along with memory deficits and dysphoric disorder [2, 3]. Plenty of studies have shown that people with PTSD are more susceptible to manifest cognitive disorder, especially in veterans [4–7]. And the person who suffered from a severe trauma and had PTSD-like symptoms during their childhood have a high risk of dementia in old age [8]. On the contrary, SSRI (Selective Serotonin Reuptake Inhibitor) treatment improves symptoms of PTSD and reduces the risk of dementia [9]. These studies strongly support that PTSD is closely linked to cognitive disorder, and therefore deserves more attention to be paid on.

Less than 1% of the AD (Alzheimer disease) cases belong to familial type due to a mutation in the APP (β-amyloid precursor protein) or PS (presenilin) 1 or PS2 [10]. More researches have focused on environmental factors that may be involved in the sporadic type of AD, which is histopathologically characterized by intraneuronal neurofibrillary degeneration of abnormally hyperphosphorylated tau and extracellular β-amyloidosis and clinically presents as dementia in humans [11]. AD is an important concern in people with physiological or psychological stress episodes, and these are at a high risk of developing this disease [12, 13], which implies causal links between stress exposure and AD neuropathological alterations. Previous studies have shown that some environmental stressors, such as cold water, heat shock, starvation, hypothermia and anesthesia, may induce tau hyperphosphorylation in the hippocampus [14–18]. Hyperphosphorylated tau loses its ability to bind and stabilize microtubule networks, and is more liable to self-aggregation into paired helical filaments (PHFs), which comprise neurofibrillary tangles (NFTs). The amount of NFTs in the brain is positively correlated with the severity of clinical dementia in AD, implicating that tau hyperphosphorylation may be involved in cognitive impairments. However, the mechanism underlying stress, especially PTSD, inducing tau hyperphosphorylation is still unclear. We here chose a consensus PTSD model - single prolonged stress (SPS) model [19] - to investigate the relationship between PTSD and tau pathology, and found that SPS-induced PTSD upregulates AKT/GSK3β and down-regulates SGK1/ERK1/2, which incongruously result in differential tau hyperphosphorylation at different sites and PTSD-like anxiety.

## RESULTS

### SPS led to PTSD-like anxiety behavior in rats

**Figure 1:**
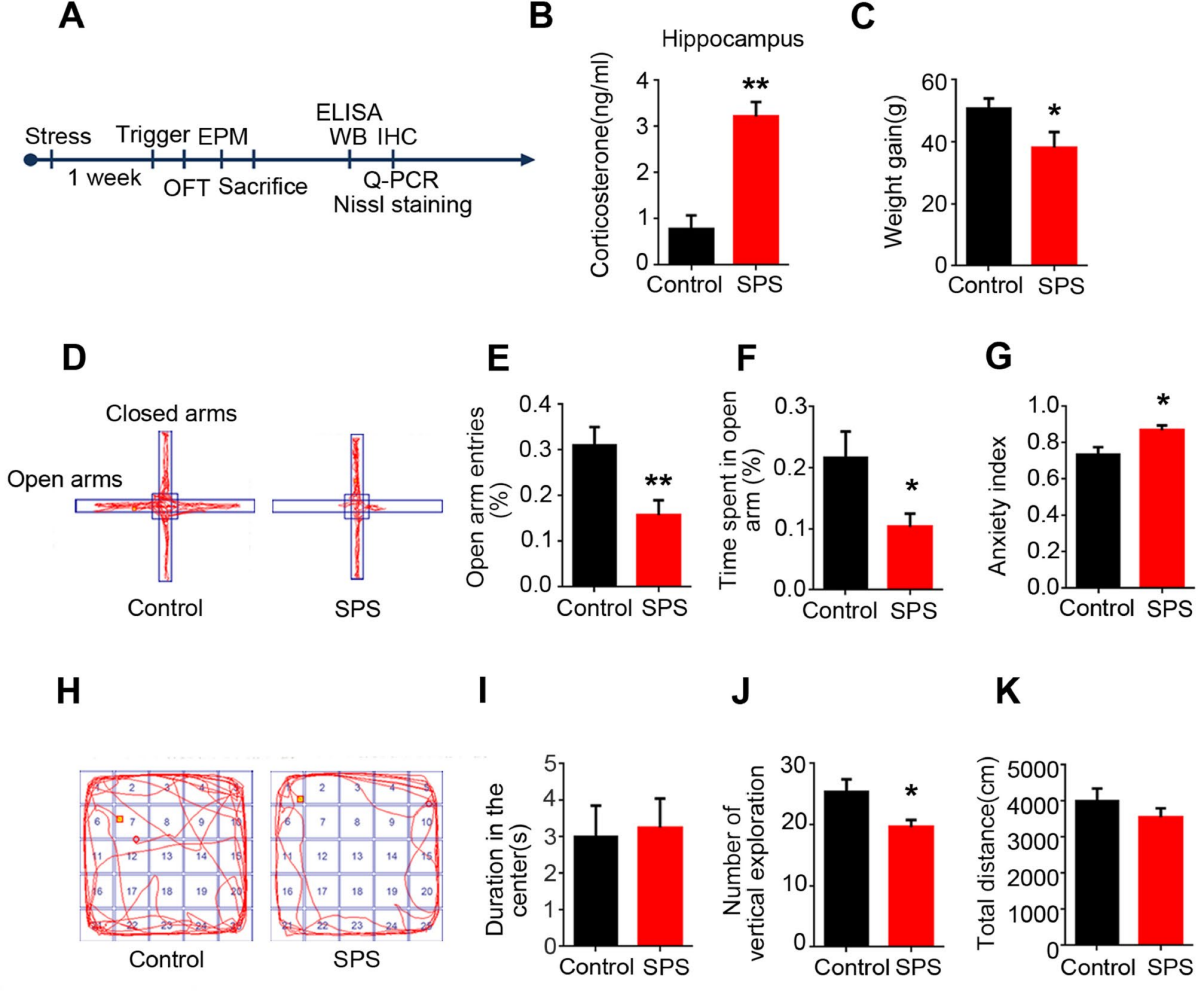
**SPS induces PTSD-like anxiety in rats.** (**A**) Time flow diagram of events in the experimental design. (**B**) Corticosterone was detected by ELISA kit. And the level of corticosterone was significantly increased in SPS-rats hippocampi. The data represent as mean ± SEM, n=3. (**C**) Rats’ weigh was measured on the first and seventh day, the data showed a lower weight gain in SPS exposed group. (**D**) The path diagrams of the elevated plus maze. (**E** and **F**) The percentage of open-arm entries and the percentage of time spent in open-arm were measured. SPS model showed significant reduction in the number of entries and time spent in the open-arm compared to control. (**G**) Anxiety index was calculated and the SPS group showed higher values. (H) The path diagrams representative of the open field test. (**I**) No significant difference in the time spent in the center. (**J**) The times of vertical exploration is significantly decreased in SPS model. (**K**) The total distance covered showed no changes compared with the control group. Behavior data show mean ± SEM, n=15, *P < 0.05, **P < 0.01 vs control.

Three stressors in single prolonged stress (SPS) paradigms were reported to induce psychological, physiological and endocrine stress respectively [19]. In the present study, we employed SPS to assess the PTSD like anxiety behavior in rats. Seven days after 2-hours restraint followed by forced swim, then by ether anesthesia, behavioral, biochemical and morphological assays were carried out (Figure 1A). ELISA data showed that SPS led to a highly significant increase in the level of corticosterone in the hippocampus of rats (Figure 1B). Body weight gain was used to assess the physical changes induced by the series of stress exposure. We found that SPS exposure group presented an obvious decrease in weight gain compared to the control group (Figure 1C). The elevated plus maze (EPM) and open field test (OFT) were performed on 8^th^ to test anxiety behaviors. In EPM (Figure 1D), it was observed that SPS rats exhibited a significant reduction in the number of entries and time spend in the opened arm compared to control (Figure 1E and 1F). The calculated anxiety index which intuitively reflects the level of anxiety, showed that SPS induced a marked increase in anxiety index. (Figure 1G). In OFT (Figure 1H), SPS induced a higher tendency of the animal to stay in the center of the maze without significant difference (Figure 1I), but there is a marked decrease in the number of vertical exploration (Figure 1J) compared to control, indicating that SPS rats presented a higher tendency of being anxious. Meanwhile, we found that the total distance remained comparable between the two groups, implying that SPS does not affect motor function (Figure 1K). All above data strongly support that SPS induced PTSD-like anxiety in rats.

### Tau hyperphosphorylation at specific sites in the hippocampus of SPS rats

**Figure 2:**
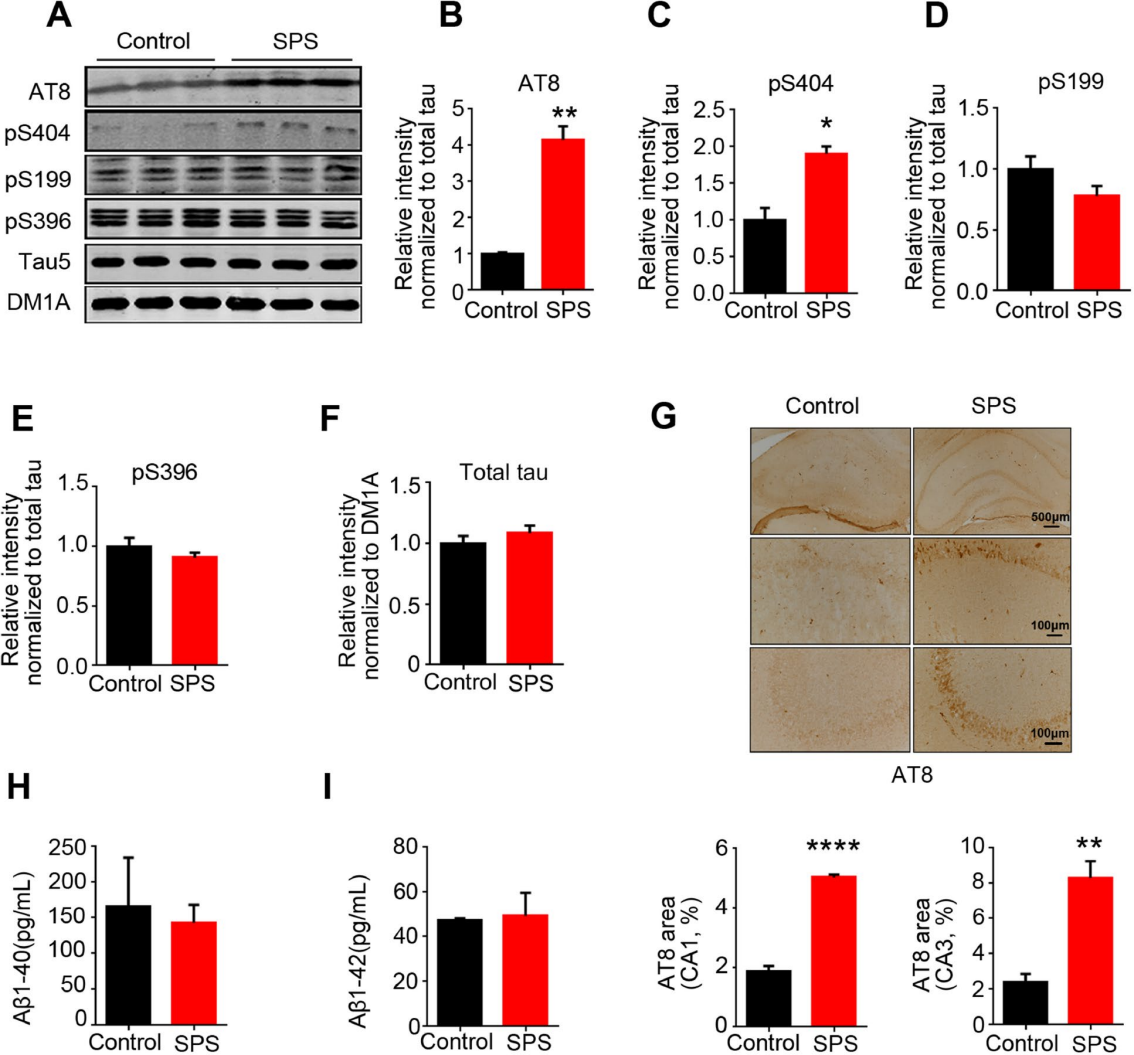
**SPS-induced PTSD leads to tau hyperphosphorylation at specific sites, but doesn’t alter Aβ1-40 and/or Aβ1-42 production.** (**A**) Hippocampal protein levels of total tau, p-tau at Ser199, Ser202/Thr205(AT8), Ser396 and Ser404 were measured by WB. (**B** and **C**) Statistical analysis showed a marked increased at AT8(Ser202/Thr205) and Ser404 in SPS rats. (**D** and **E**) Statistical analysis of the level of pS199 and pS396 showed no significant differences between the two groups. (**F**) Total tau (tau5) normalized by DM1A. (**G**) Immunohistochemistry showed the increased distribution of AT8 compared to control (upper row scale bar=500μm, middle and lower row scale bar=100μm). (**H** and **I**) Measurement of Aβ1-40 and Aβ1-42 by ELISA kits in hippocampus. All data represent mean ± SEM, n=3, *P < 0.05, **P < 0.01 vs control, ****P<0.0001 vs control.

Accumulating evidence suggests that PTSD is a potential risk factor for Alzheimer’s disease (AD) [20–22]. Hyperphosphorylation of tau is one of typical neuropathological alterations of AD, which may be involved in behavioral dysfunction [23–25]. To investigate whether SPS-induced PTSD is associated with tau hyperphosphorylation, we examined the level of p-tau at different AD-relevant sites in the hippocampus of rats by immunoblotting analysis (Figure 2A). As expected, we observed a marked increase in tau phosphorylation at Ser202/Thr205 (AT8) and Ser404 in the hippocampus of SPS rats compared to control (Figure 2B–2C). Interestingly, the level of phosphorylation of tau at Ser199 and Ser396, two other important AD-relative sites, were not significantly different between the two groups (Figure 2D–2E). The levels of phosphorylated tau (p-tau) at above sites were normalized to total tau developed with Tau5, which was not altered by SPS (Figure 2F). We also carried out immunohistochemical assay and found a very strong staining with AT8 (p-tau at Ser202/Thr205) in the hippocampal CA1 and CA3 regions of SPS rats (Figure 2G). These data suggest that SPS induces selective specific sites tau hyperphosphorylation in the hippocampus of rats.

Besides tau pathology, Aβ-containing senile plaque is the other typical neuropathological change in AD. Previous study showed that PTSD-Like induction elevates Aβ level [26]. We therefore investigated the effect of SPS on Aβ production. Surprisingly, ELISA data showed that SPS has no effect on the level of both Aβ1-40 and Aβ1-42 (Figure 2H–2I), implying that this discrepancy may result from different stimulus consequence on the Aβ toxicity.

### PTSD-like induction altered the activities of tau related protein kinases

**Figure 3:**
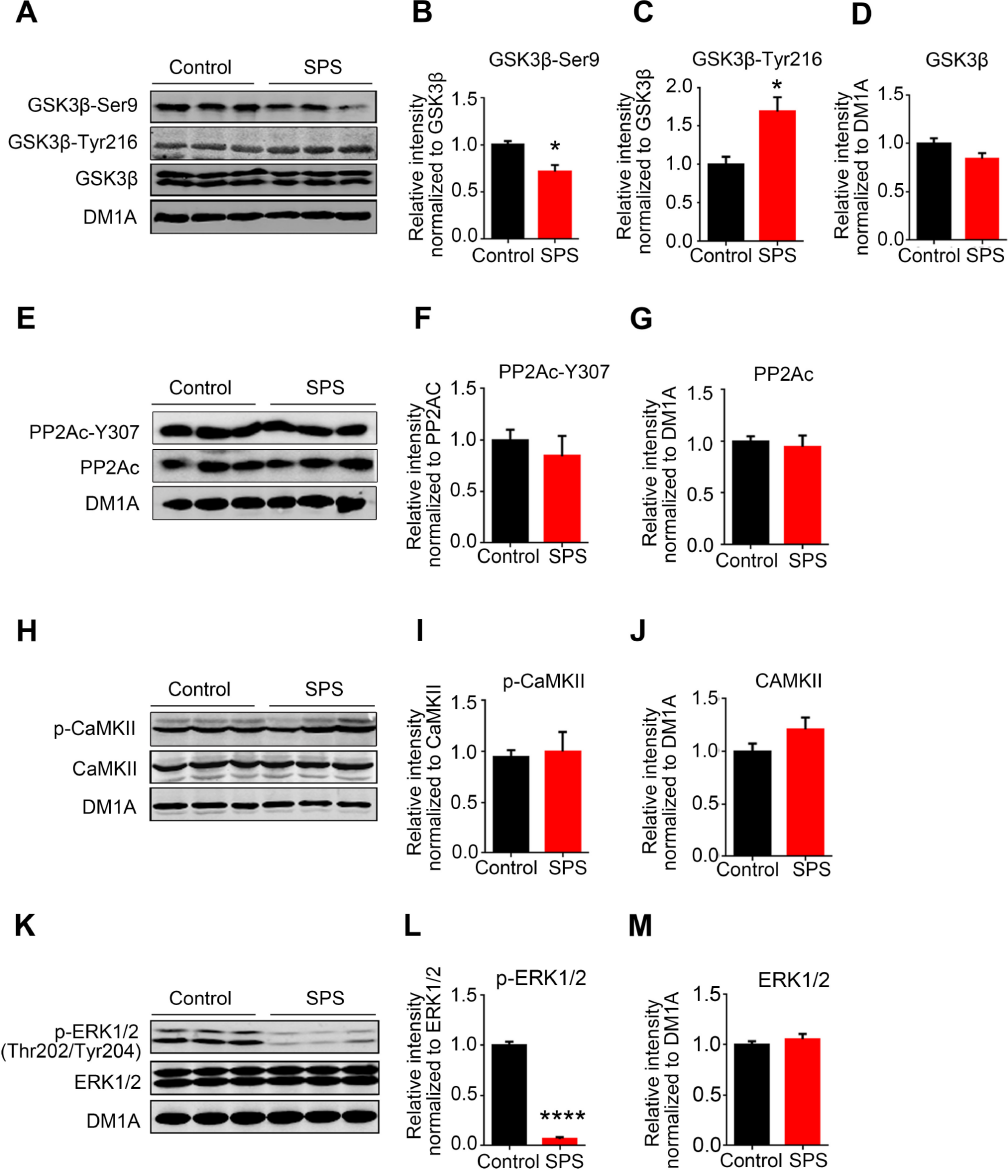
**SPS modulates tau-related kinases not phosphatase.** (**A**) The level of total and phosphorylated GSK3β at Ser9 and Tyr216 were measured by WB. (**B** and **C**) SPS rats showed a decreased phosphorylation of GSK3β at Ser9 (B) and a significant increase at Tyr216 (**C**). (**D**) Total GSK3β didn’t change compared with control. (**E**, **F** and **G**) WB showed no changes in the levels of both PP2Ac and phosphor-PP2Ac-Y307. (**H**, **I** and **J**) CaMKII and p- CaMKII were detected by Western blot and showed no significant difference. (**K**, **L** and **M**) The level of p-ERK1/2 was significantly decreased in SPS rats and total ERK1/2 showed no changes compared with control. All data represent mean ± SEM, n=3, *P < 0.05, **P < 0.01 vs control, ****P<0.0001 vs control.

To explore how tau is differentially hyperphosphorylation in PTSD-like rats, we detected several kinases and phosphatase closely related to tau phosphorylation. Glycogen synthase kinase-3 beta (GSK-3β) - also known as human tau protein kinase [27]. Phosphorylation of GSK-3β is regarded as the main regulatory mechanism on GSK-3β activity in physiological and some pathological conditions. Phospho-GSK-3β at Tyr216 is an activated form of GSK-3β while phospho-GSK-3β at Ser9 is an inactivated form of GSK-3β [28]. In the present study, western blotting analysis showed that SPS-induced PTSD did not change the level of total GSK-3β (Figure 3A and D), but markedly decreased the expression of phosphorylated-GSK-3β at Ser9 (Figure 2B) and increased the expression of phosphorylated-GSK-3β at Thr216 in the hippocampus as compared to control (Figure 2C), supporting that SPS upregulates GSK-3β activity. However, protein phosphatase 2A (PP2A), as a major regulator of tau phosphorylation, showed no significant changes in neither the total level of its catalytic subunit (PP2Ac) nor its phosphorylated (inactivated) form at tyrosine 307 (PP2Ac-Y307) between the two groups (Figure 3E–3G), indicating no alteration of PP2A activity in SPS rats. These data suggest that PTSD-like induction upregulates GSK-3β activity, which might contribute to the observed tau hyperphosphorylation.

Actually, it was observed in the current study that tau was highly phosphorylated at Ser202/Thr205 (AT8) and Ser404 but not at Ser199 or Ser396 in the hippocampus of SPS rats. To address this differential tau phosphorylation pattern, we detected other tau-related protein kinases including CaMKII and ERK1/2. Western blotting result showed that PTSD-like induction did not alter the level of phosphorylated-CaMKII, total CaMKII and total ERK1/2 (Figure 3H–3J, 3K and 3M), but extremely decreased the expression of phosphorylated-ERK1/2 when compared to control (Figure 3K and 3L), indicating that SPS downregulates ERK1/2 activity. A previous study has reported that ERK1/2 activation induces hyperphosphorylation of tau at Ser199 and Ser396 [29]. Therefore, we speculate that downregulation of ERK1/2 might mask or at least attenuate the phosphorylation effect of GSK-3β at Ser199 and Ser396 sites leading to tau hyperphosphorylation at other specific sites (Ser202/Thr205 and Ser404) but not at Ser199 and Ser396 in the hippocampus of SPS rats.

### Downregulation of AKT activity and SGK1 level in the hippocampus of SPS rats

**Figure 4:**
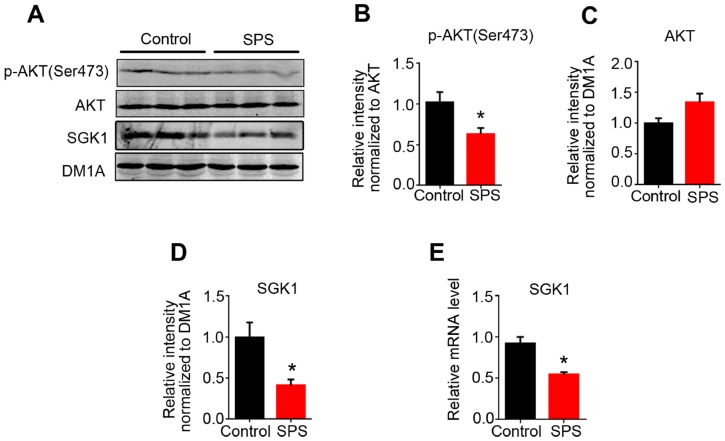
**SPS downregulates the activity of AKT and SGK1 expression level.** (**A**) WB results showed the level of AKT, p-AKT at Ser 473, SGK1. (**B** and **C**) The level of p-AKT was decreased, but total AKT protein level didn’t change. Protein (**D**) and mRNA (**E**) levels of SGK1 were markedly decreased in SPS rats compared to control. All data represent mean ± SEM, n=3, *P < 0.05 vs control.

GSK3β is a target of AKT and the serum and glucocorticoid inducible kinase (SGK1). Phosphorylation of GSK3β at Ser9 by AKT or SGK1 inhibits GSK3β activity [30, 31]. Moreover, both AKT and SGK1 are associated with stress [32, 33] and the level of SGK1 is usually increased within a short period of time under acute stress and also decreased in PTSD patients [34]. To evaluate whether the increased GSK3β activity seen in the hippocampus of SPS rats is a reflection of both AKT and/or SGK1 alterations, we next detected the expression of AKT and SGK1. We found that SPS induced a significant decrease in the expression of phosphorylated-AKT at Ser473 in the hippocampus as compared to control, while the level of total AKT showed no significant difference between the two groups (Figure 4A–4C), suggesting PTSD-like induction leads to AKT inactivation. Data from western blotting and q-PCR showed that the level of both SGK1 protein and mRNA in the hippocampus of SPS rats was markedly decreased when compared with the control (Figure 4A, 4D and 4E). These findings insinuate that the increased GSK3β activation in SPS rats may at least in part be probably due to downregulation of AKT activity and SGK1 level.

### Overexpression of SGK1 induced ERK1/2 activation and tau hyperphosphorylation at Ser199 and Ser396 in HEK293/tau cells

**Figure 5:**
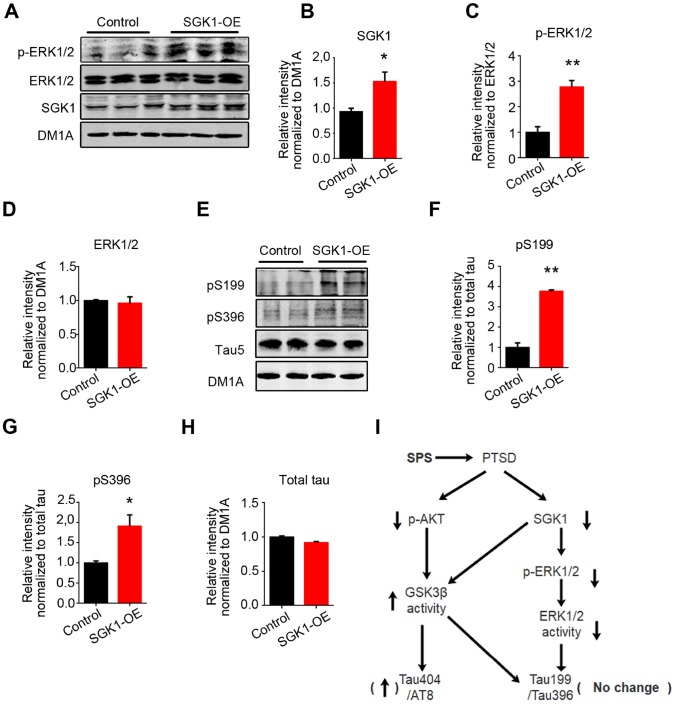
**SGK1 overexpression upregulates the p-ERK1/2 and increases tau hyperphosphorylation at Ser199 and Ser396 in HEK293/tau cells.** (**A**) HEK293/tau cells were transfected with SGK1 plasmid, and WB was performed to detect p-ERK1/2, ERK1/2 and SGK1. (**B**) Quantification showed the significantly increased level of SGK1. (**C** and **D**) SGK1 overexpression increased phosphorylation of ERK1/2. (**E**) Overexpression of SGK1 evidently increased the level of pS199 and pS396 compared with control. (**F**–**H**) Quantitative analysis for blot in E. (**I**) Model showing how downregulated SGK1 and AKT contribute to PTSD. All data represent mean ± SEM, n=3, *P < 0.05 vs control, **P < 0.01 vs control.

SGK1 has been shown as an activator of ERK1/2 [35]. To further determine whether SGK1 regulates ERK1/2 activity and mediates tau phosphorylation, we transfected HEK293/tau with SGK1 plasmids and performed western blotting. The result revealed that overexpression of SGK1 (Figure 5A and 5B) induced a significant increase in the expression of phosphorylated-ERK1/2 (activated form) in the hippocampus as compared to control, while the total ERK1/2 showed no significant difference between the two groups (Figure 5A, 5C and 5D). As expected, the level of tau phosphorylation at Ser199 and Ser396 in HEK293/tau/SGK1 cells was evidently increased as compared to control (Figure 5E–5G), while the level of total tau recognized by Tau 5 had no difference between the two groups (Figure 5E and 5H). Meanwhile, we found that overexpression of SGK1 markedly increased the expression of phosphorylated-GSK-3β at Ser9 (Supplementary Figure 1A–1C) and induced a marked decrease in tau phosphorylation at Ser202/Thr205 (AT8) as compared to control (Supplementary Figure 1A and 1D), while total tau had no change (Supplementary Figure 1E), supporting that GSK activation is response for tau phosphorylation at Ser202/Thr205 (AT8) in SPS rats. These findings imply that SGK1 deficiency might lead to at least decreased activity or even complete inactivation of ERK1/2 which in turn lead to a decrease in tau phosphorylation at Ser199 and Ser396.

Taken together, all above data suggest that PTSD-like induction downregulates SGK1-related ERK1/2 activity and upregulates AKT/SGK1-related GSK3β activity, finally resulting in tau hyperphosphorylation at Ser202/Thr205 (AT8) and Ser404 but not at Ser199 or Ser396 epitopes in the hippocampus of SPS rats (Figure 5I).

### No neuronal loss or synaptic dysfunction in SPS rats

**Figure 6:**
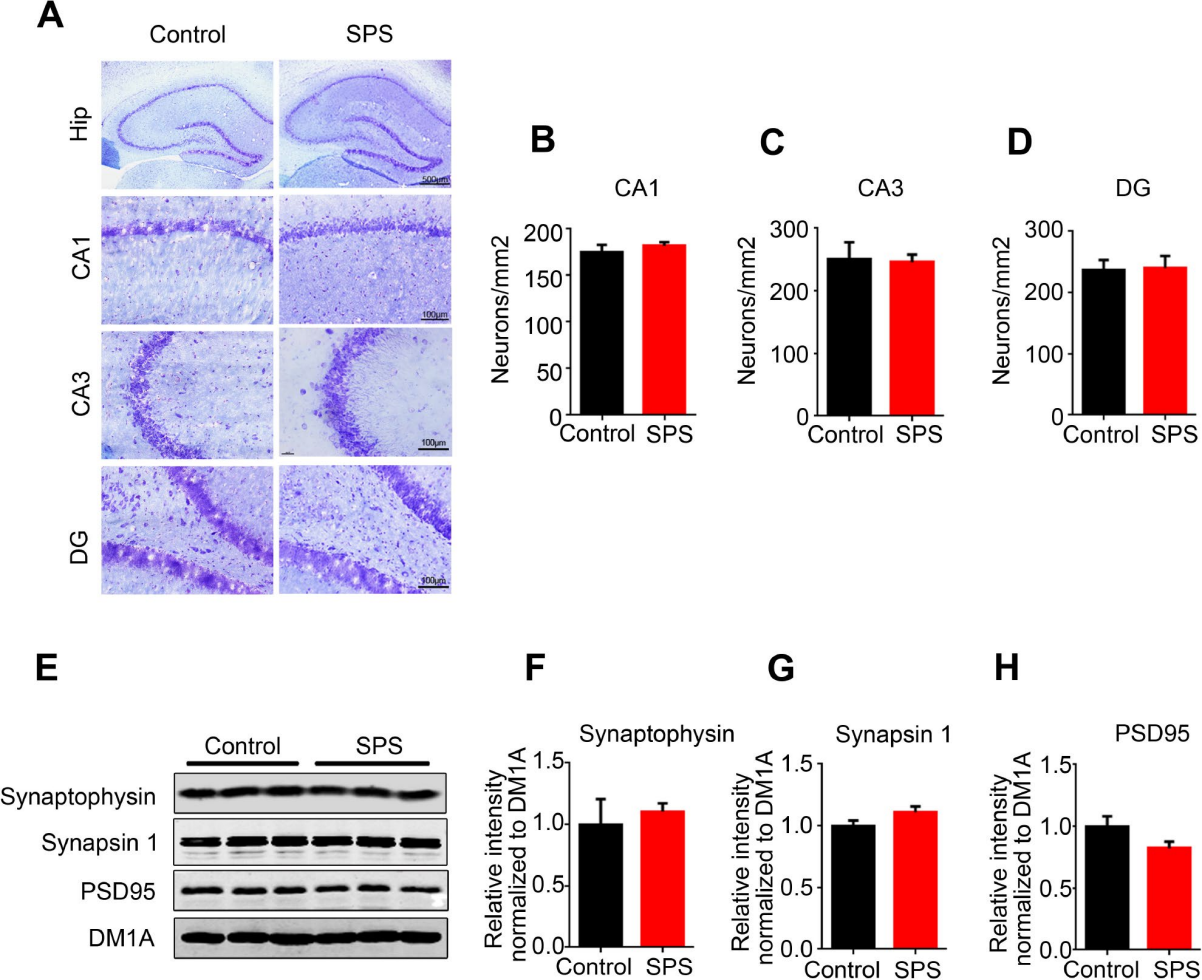
**SPS does not influence neuronal and/or synaptic density.** (**A**) Hippocampal sections were stained by Nissl staining to assess neuronal density (upper row scale bar=500μm, middle and lower row scale bar=100μm). (**B**–**D**) Quantitative analysis was performed, and SPS didn’t induce the neuronal death in CA1(**B**), CA3(**C**) and DG(**D**). (**E**–**H**) WB analysis showed no significant changes in the level of synaptophysin, synapsin1 and PSD95. Data are presented as mean ± SEM, n=3.

Long-term PTSD patients may suffer from cognitive disorder in old age. The loss of neurons and synaptic deficiency are the main causes of cognitive impairments. To study whether PTSD induction resulted in neuronal loss in SPS rats, we performed Nissl staining to assess the neuronal density. We found that there was no significant difference in term of neuronal density in the CA1, CA3 and dentate gyrus of hippocampus of SPS rats as compared to control (Figure 6A–6D), indicating no neuronal loss. Normal synaptic function relies on the stable expression of synaptic proteins, such as PSD95 in the post-synapse and synapsin-1 in the pre-synapse. Therefore, we evaluated the expression levels of these synapse-associated proteins by using Western blotting. The result showed no obvious difference in the expression of synaptophysin, synapsin-1 and PSD95 between SPS rats and control ones (Figure 6E–6H). Immunofluorescence with synapsin 1 showed same result (Supplementary Figure 2). Together these data indicate that the cause of PTSD-like behavioral disorder observed in this study is not due to loss of neurons and/or synaptic dysfunction and therefore might need further investigation.

### No alterations in both p-tau and related protein kinases in prefrontal cortex of SPS-rats

**Figure 7:**
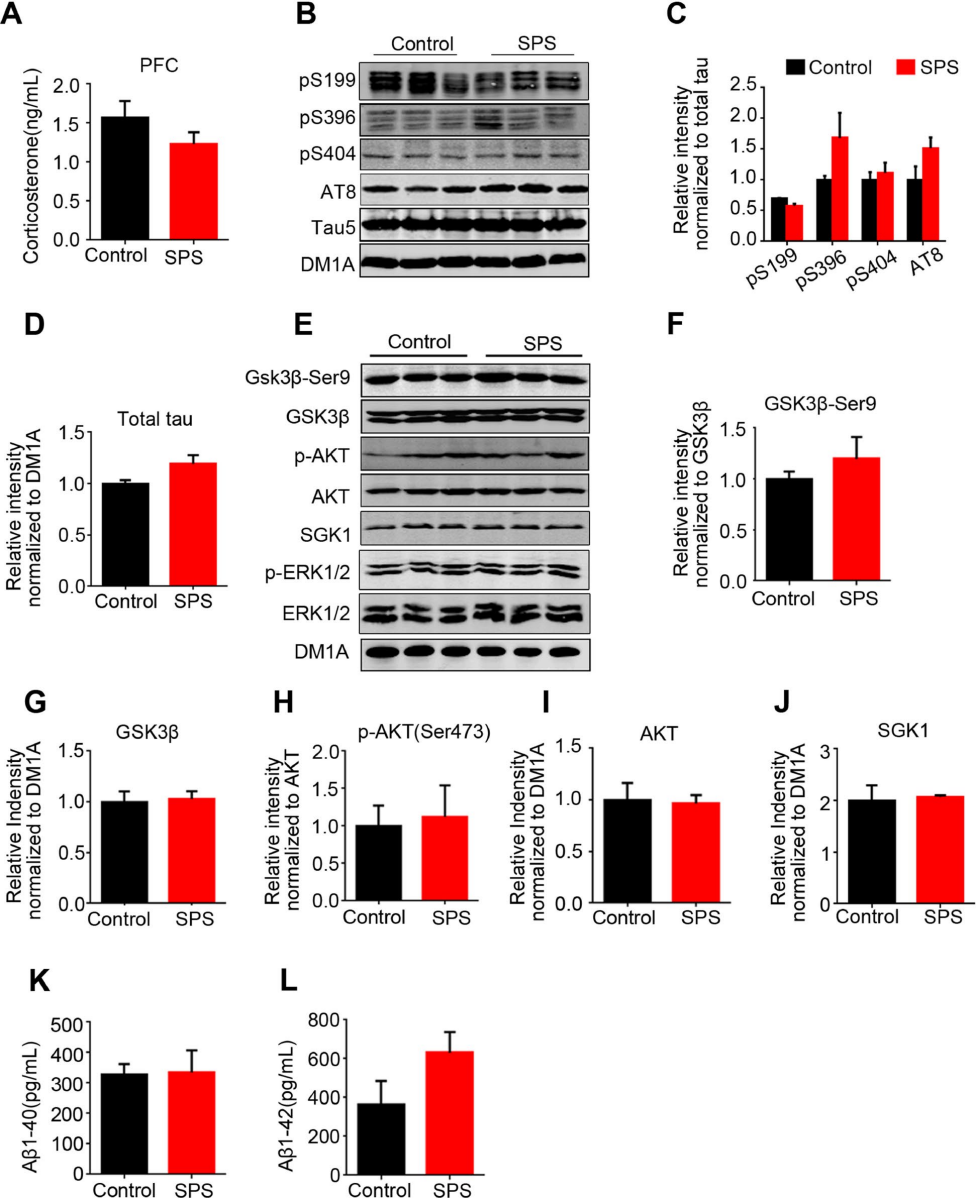
**No change of tau and/or tau-related kinases in the prefrontal cortex of SPS rats.** (**A**) The level of corticosterone wasn’t significant different compared with control in prefrontal cortex. (**B**–**D**) WB protein bands showed an increase tendency of tau, but no statistical significance. (**E**–**J**) tau-related kinase detected by WB showed no changes between the two groups. (**K** and **L**) SPS-induced PTSD didn’t alter Aβ1-40 and Aβ1-42 production in the prefrontal cortex. All data represent mean ± SEM, n=3.

In patients with AD, abnormally hyperphosphorylated tau proteins are preferentially deposited in vulnerable brain regions, including cortex and hippocampus, which is essential for learning and memory formation [36]. In the current study, we also investigated the effect of SPS on tau phosphorylation and related protein kinases in the prefrontal cortex (PFC). Firstly, the level of corticosterone in PFC was evaluated and the findings showed no significant difference between SPS rats and control (Figure 7A). Next, we performed western blotting and found out that SPS neither affected tau phosphorylation status nor total tau level in PFC (Figure 7B–7D). Moreover, all above-mentioned tau-related kinases, including GSK-3β, phosphorylated GSK-3β, AKT, phosphorylated AKT, SGK1, had no significant changes in the PFC of SPS rats when compared to control ones (Figure 7E–7J). Finally, Aβ production was quantified, and the results revealed no alterations in the levels of both Aβ1-40 and Aβ1-42 in PFC (Figure 7K and 7L). Together, these findings suggest that PTSD-like induction does not affect tau phosphorylation or Aβ production in PFC of SPS rats.

## DISCUSSION

People with PTSD are more likely to develop AD as they age [7]. Previous studies have shown that single-session trauma exposure elevates levels of CSF Aβ and repeated stress exposure leads to chronic increase in tau phosphorylation in the hippocampus [26, 37]. However, the specific molecular interconnections between PTSD and these neuropathological alterations is still not well known. In the current study, we mainly investigate the mechanism underlying the effect of SPS-induced PTSD on tau hyperphosphorylation at AD-relevant sites in different brain regions of rats. We found that wild-type rats displayed a typical PTSD-like anxiety behavior after SPS, and showed a decreased or slow weight gain which is probably due to anxiety induced poor appetite and with no anti-PTSD treatment. Furthermore, SPS upregulates AKT/SGK1 related GSK3β activity and downregulates SGK related ERK1/2 activity, finally resulting in differential tau hyperphosphorylation at Ser202/Thr205 (AT8) and Ser404 but not at Ser199 or Ser396 in the hippocampus of these rats.

Fear and anxiety, which are thought to be essential to the etiology of PTSD in humans [38], usually serve as main reference symptoms in modelling the construction of PTSD in animals. SPS pattern employed in the present study obviously induced an anxiety behavior in rats suggesting the induction of PTSD-like symptoms, and the level of corticosterone in the hippocampus was markedly increased in the SPS rats. The level of corticosterone is rapidly increased at the alarm stage of stress, while it is also increased in AD [39]. During the late stage of stress, corticosterone may be at a low level due to the increase of its exhaustion. Our data suggest that SPS-induced PTSD has not yet developed into the late stage. Previous study reported that PTSD-like induction elevates Aβ [26]. We here found that at this stage SPS does not induce an increase in the Aβ level in both the hippocampus and prefrontal cortex, and this might be due to the elevated corticosterone reflecting its increased Aβ cleaving activity [26].

In the present study, following PTSD-like induction, we found tau hyperphosphorylation in the hippocampus of SPS rats. This is consistent with previous studies that showed stress exposure acts as a risk factor for tau pathology [40]. Neuroendocrine changes are one of the responses that occur during stress exposure and PTSD is an advanced stage of stress. Moreover, neuroendocrine responses are always regarded to be associated with neuropathological changes in AD patient with PTSD. Robert A. Rissman et al. reported that corticotropin-releasing factor receptor 1 (CRFR1) regulates stress-induced tau phosphorylation in the murine hippocampus [41, 42]. An unexpected and potentially significant finding of the present study is that PTSD-like induction led to a differential tau hyperphosphorylation pattern at specific sites including Ser202/Thr205 (AT8) and Ser404 but not at Ser199 or Ser396 in the hippocampus. To address the mechanism underlying this pattern of tau phosphorylation, we detected tau-related kinases and phosphatase, and interestingly found that SPS induced GSK-3β activation and ERK1/2 downregulation but has no effect on the activities of CaMKII and PP2A, insinuating that GSK-3β activation contribute to tau hyperphosphorylation mainly at Ser202/Thr205 (AT8) and Ser404 while ERK1/2 deficiency masked or attenuated GSK-3β-mediated tau hyperphosphorylation at Ser199 and Ser396 in the hippocampus of SPS rats. Moreover, it was reported that ERK1/2 is involved in tau phosphorylation at both Ser199 and Ser396 [43], and GSK-3β, even though it phosphorylates both primed and non-primed sites, preferentially phosphorylates sites that are primarily phosphorylated by other kinases with more efficiency (50 folds) [44, 45]. This further suggests that the probable lack of ERK1/2 priming effect on Ser199 and Ser396 sites might in at least in part explain the non-GSK-3β mediated hyperphosphorylation at these sites. Actually, inhibition of PP2A induced tau hyperphosphorylation under hypothermic condition, whereas major tau kinases like JNK and MAPK are not activated [17]. Tau hyperphosphorylation in brain slices is probably attributable to the direct inhibition of PP2A by low temperatures rather than to kinase activation. However, stress response is a complicated process which might be caused by different stimulus and induces different alterations in the expression and posttranslational modification of proteins. Our failure to discern SPS effect on total and/or phosphorylated PP2A-c levels is not necessarily indicative of a lack of phosphatase involvement under these conditions.

GSK3β is a target of stress-related AKT and SGK1 [30]. The activation of the PI3K/AKT pathway seems to predominantly downregulate GSK-3β activity via its Ser9 phosphorylation, while the activation of AKT is associated with increased phosphorylation of its Ser473 and Thr308 residues [46]. Here we found that PTSD-like induction led to a significant decrease in both the activated (pS473) form of AKT and total SGK1 levels, which might account for GSK-3β activation in the hippocampus of SPS rats. Probably, Fyn, a protein kinase of the Src tyrosine kinase family, may directly phosphorylate GSK-3β at Tyr216 and thereby increasing its activity, which hypothesis should be further investigated and clarified. SGK1 is quickly increased under acute stress and decreased in PTSD [34], and this is consistent with the present findings. Meanwhile, as a kinase of ERK1/2, SGK1, when overexpressed in HEK293 tau cells, it induced an increase in the level of activated form of ERK1/2 resulting in tau hyperphosphorylation at Ser199 and Ser396. This further supports that GSK3β and ERK1/2 differentially regulate tau phosphorylation at Ser202/Thr205 and Ser404 but not at Ser199 or Ser396 in the hippocampus of SPS rats, suggesting a probable priming effect of ERK1/2 on Ser199 and Ser396 for GSK-3β phosphorylation.

Normal synaptic function relies on the stable expression of synaptic proteins, such as synaptophysin, synapsin-1 and PSD95. Our findings revealed that SPS-mediated PSTD-like induction does not affect the expression levels of these synapse-associated proteins, and together with Nissl staining result support that the cause of PTSD-like behavioral disorder is not due to the loss of neurons and/or synaptic dysfunction. Besides hippocampus, the other brain regions that are also associated with either AD or PTSD is the cortex [36]. In the current study, there were no significant alterations in both p-tau and its related protein kinases in PFC of SPS rats, implying that PTSD-like induction does not affect tau phosphorylation in PFC of rats.

Besides anxiety, cognitive impairments might be also observed in people with PTSD, especially as they age. The endogenous opioid system is activated by PTSD, which is associated with the pathogenesis of AD, including increased Aβ toxicity and tau pathology, supporting that opioid system dysfunction is involved in cognitive impairment in PTSD accompanied by AD [21]. It is worth further studying cognitive alteration and its mechanism underlying PTSD models.

In summary, we have found in the present study that PTSD-like induction leads to tau hyperphosphorylation at specific AD-relevant sites in the hippocampus of SPS rats, which as a result of incongruous action between AKT-related GSK-3β activation and SGK1-related ERK1/2 inactivation resulted in the phosphorylation of tau at Ser202/Thr205 and Ser404 but not at Ser199 or Ser396. Given that SPS does not affect Aβ production and synaptic function under this circumstance, reduction of tau hyperphosphorylation rather than Aβ toxicity might be a major pharmacological interference for treating AD patient with PTSD.

## MATERIALS AND METHODS

### Plasmids, chemicals and antibodies

The SGK1 (NM_005627) plasmid encoding Ubi-MCS-3FLAG-CBh-gcGFP-IRES-puromycin was generated in our laboratory. Plasimid was prepared with endotoxin-free plasmid extraction kit (Tiangen). HighGene transfection reagents were from ABclonal Biotechnology Co., Ltd. Culture medium was from Gibco BRL (Gaithersburg, MD, USA). Antibodies used in this study are listed in Table 1.

**Table 1:** Antibodies employed in this study.

**Antibody**	**Specificity**	**Type**	**Dilution**	**Source**
Tau5	Total tau	mAb	1:1000 for WB	Abcam
AT8	Phosphorylated at Ser202/Thr205	mAb	1:1000 for WB 1:200 for IHC	Thermo Fisher
pS199	Phosphorylated at Ser199	pAb	1:1000	Thermo Fisher
pS396	Phosphorylated at Ser396	pAb	1:1000	Abcam
pS404	Phosphorylated at Ser404	pAb	1:1000	SAB
SGK1	Antibody for serum and glucocorticoid-regulated kinase 1	pAb	1:1000	Abcam
p-ERK1/2	ERK1/2 phosphorylated at Thr202 and Tyr204	pAb	1:1000	CST
ERK1/2	Total ERK1/2	pAb	1:1000	CST
CaMKII	Ca^2+^/calmodulin-dependent protein kinase II	pAb	1:1000	Thermo
p- CaMKII	CaMKII phosphorylated at Thr286	pAb	1:1000	CST
PP2Ac	Protein Phosphatase 2Ac	pAb	1:1000	CST
PP2Ac-Y307	PP2Ac phosphorylated at Tyr307	pAb	1:1000	Merck Millipore
GSK3β	Glycogen synthase kinase 3 beta,	pAb	1:1000	SAB
GSK3β-Ser9	GSK3β phosphorylated at Ser9	pAb	1:1000	CST
GSK3β-Tyr216	GSK3β phosphorylated at Tyr216	pAb	1:1000	Abcam
AKT	Total AKT(PKB)	pAb	1:1000	CST
p-AKT(Ser473)	AKT phosphorylated at Ser473	pAb	1:1000	CST
PSD95	Post synaptic density protein	pAb	1:1000	CST
Synapsin-1	Synapsin Ia and synapsin Ib	pAb	1:1000	Merck Millipore
Synaptophysin	Major synaptic vesicle protein p38	pAb	1:1000	Abcam
DM1A	Recognizing alpha-tubulin	mAb	1:1000	Sigma

mAb Monoclonal antibody, pAb Polyclonal antibody, WB Western blot, IHC Immunohistochemistry.

### Animals

Thirty male Sprague-Dawley rats (8 weeks old, 250±30g) bred at the Experimental Animal Central of Tongji Medical College were used in this research. Animals were housed five per cage with accessible food and water under a 12: 12 light-dark cycle with the light on from 7:00 am to 7:00 pm. All animal experiments were performed according to the “Policies on the Use of Animals and Humans in Neuroscience Research” revised and approved by the Society for Neuroscience in 1995.

### Single prolonged stress (SPS)

The procedure was carried out as a classic method [19]. Rats were restrained in a plastic bottle for 2 hours, then were put into a barrel with 2/3 height of clean water (height: 60cm, diameter: 50cm) to force them to swim immediately until they were exhausted. After that, rats were permitted to take a rest for 20 mins and then anesthetized by the ether until they lose consciousness. Rats were taken back to their cages and fed routinely. Meanwhile, the control group rats were kept in cages without any stimulus. After seven days, a 20 mins restraint trigger was given, and the next day behavior tests were performed.

### Open field test (OFT)

OFT is used for evaluating the tension of experimental animals, as described earlier [47]. In the quiet movement, each rat was placed in a wooden box (100 ×100×50cm) which was equally divided into 25 sectors, keeping the head in the same direction. Each rat was permitted to freely explore the new environment for 5 mins, and a tracking camera recorded the total distance covered by the animal. The center duration (%) and vertical activity were used to scale the anxiety. After each experiment, 75% alcohol was used to sweep the box before the next animal was taken in.

### The elevated plus maze (EPM)

The EPM was utilized to determined anxiety behavior and was performed as previously described [48]. The apparatus consists of two opposed closed arms (50×10×40cm) and two opposed opened arms (10cm×50cm), placed on a 40 cm height platform. In the dim light, each of these rats was allowed to freely move around for 5 minutes. The distance open arm, distance closed-arm, duration open arm, duration closed arm, open arm entry and closed arm entry were recorded by a tracking camera. The anxiety index was calculated as previously reported: 1 − [(time spent in open arm/total time on the maze)/2 + (number of entries to the open arms/total exploration on the maze)/2] [49].

### Immunohistochemistry

Rat brains were perfused with normal saline followed by 4% paraformaldehyde and processed for immunohistochemistry. Briefly, brains were cut into 30 μm sections and rinsed in PBS. After permeabilization with washing buffer containing (0.3% H_2_O_2_, 0.5% Triton-100), slices were blocked with 3% BSA and incubated overnight with primary antibody at 4°C. Subsequently after washing, secondary antibody was employed. Finally, dyed with DAB (Zsbio Commerce Store, Beijing, China). Images of p-tau (AT8) positive cells in the hippocampus were digitalized using a microscope (Nikon) fitted with ×4 and ×20 objective lens.

### Nissl staining

The frozen sections were mounted on the slide, and air-dried at room temperature for 24h. Toluidine blue (Wuhan Goodbio technology CO, LTD, China) was used to stain and increasing gradient of alcohol (75%, 85%, 95%) were used to dehydrate and decolorize. The slides were cleared with dimethylbenzene for 10 min twice, then mounted and images were taken using light microscope (Nikon). Image-Pro Plus software was used to analyze cell numbers. The areas covered in regions of the hippocampus were measured and calculated in three brain sections per animal.

### HEK293/tau cells culture

HEK293/tau cells which were the human embryonic kidney 293 cells stably transfected with the longest human tau (tau441) were cultured in Dulbecco’s modified Eagle’s medium (Gibco) in the presence of 200 mg/mL G418 containing 10% fetal bovine serum Gibco BRL, Gaithersburg, MD, USA). The cells were cultured in a humidified incubator aerated with 95% air and 5% CO_2_ at 37 °C. Cells were seeded into 6-wells cultured plates and incubated for 24 h prior to transfection. Each well was transfected with the mixture containing a total of 3μg plasmids and 6μl HighGene transfection reagents (ABclonal) according to the manufacturer’s protocols. Cells were collected in subsequent experiments 48h post transfection.

### RNA extraction and real-time PCR

Total RNA from the cells was extracted by TRIzon Reagent method (CWBIO, WUHAN). First-strand complementary DNA (cDNA) was synthesized from a total of 50mg RNA using the ReverTra Ace qPCR RT kit (TOYOBO, USA). Quantitative polymerase chain reaction (PCR) was performed in a standard PCR reaction mixture prepared in duplicate using an Applied Biosystems 7900 Prism Real-Time PCR system and SYBR Premix Ex Taq (TaKaRa, Dalian, Japan) in accordance with the manufacturer’s protocol. Quantitative PCR primers were as follows: sense: 5′-CTGCAATGTGCCTTTTCTGA-3′, antisense: 5′-ATGCTTCCCTCAAGCATCTG-3′

### Western blotting

Cells samples were incubated with a buffer containing RIPA low lysis buffer and protease inhibitor for 10 mins and collected. The rats’ hippocampi were rapidly removed and were homogenized at 4°C using a buffer containing 50mmol/L Tris-HCl, pH 7.4, 150 mmol/L NaCl, 10 mmol/L NaF, 1 mmol/L Na_3_VO_4_, 5 mmol/L EDTA, 2 mmol/L benzamidine, and 1 mM PMSF. After centrifugation of the tissue homogenates at 12000 rpm/min, the supernatants were collected. Protein concentrations were quantified by Bicinchoninic acid (BCA) protein kit (Pierce, Rockford, IL, USA), a final concentration of β-mercaptoethanol (BME) and bromophenol blue (3:1) were added. The proteins in the extracts were separated by 10% SDS-PAGE and transferred to nitrocellulose membrane. The membranes were incubated with primary antibodies (see Table 1) overnight at 4°C. Then three washes with TBST, the membranes incubated with the secondary antibody at room temperature for 1 h. Odyssey Infrared Imaging System was used to visualized immunoreactive bands. Bands intensity readings were obtained using ImageJ software.

### ELISA quantification of corticosterone and Aβ

The rat CORT (corticosterone) ELISA kit and rat Aβ1-40 and 1-42 ELISA kit were utilized (96T, Elabscience). Brain tissues were cut into small pieces and washed thoroughly with PBS to remove excess blood, then homogenized in PBS (tissue weight: PBS volume=1:9) with a glass homogenizer on ice. Following the instruction of the manufacturer, wells were added with the standard or sample (100μl) and incubated at 37°C as required. Then immediately after removing the liquid, each well was incubated with biotinylated detection Ab working solution for 1 hour at 37°C. Samples were then washed 3 times and incubated with HRP conjugate working solution for 30 minutes at 37°C. Each well was washed 5 times and incubated with substrate reagent in dark room, until the color changed. Finally, stop solution was added to stop the reaction. Plates were read using a micro-plate reader set at 450 nm.

### Statistical analysis

Experimental values were obtained from at least three independent experiments with a similar pattern and expressed as means. Data were expressed as mean ± SEM and analyzed using GraphPad Prism7.0 software (GraphPad Software). Differences with p<0.05 were considered significant.

### Ethics

No humans were used in this research. All animal experiments were approved by the Animal Care and Use Committee of Huazhong University of Science and Technology, and performed in compliance with the National Institutes of Health Guide for the Care and Use of Laboratory Animals.

Aβ: β-amyloid; APP: amyloid precursor protein; GK1: Serum and glucocorticoid inducible kinase; GSK-3β: Glycogen synthase kinase-3 beta; PP2A: Protein phosphatase 2A; PS1: Presenilin 1; PS2: Presenilin 2 OFT: Open field test; EPM: Elevated plus maze; HEK293T: Human embryonic kidney 293; PCR: Polymerase chain reaction; PFC: Prefrontal cortex; SSRI: Selective serotonin reuptake inhibitor; CRFR1: Corticotropin-releasing factor receptor 1.

## Supplementary Material

Supplementary Figures
