# Supplementary material for: GSK-3β and ERK1/2 incongruously act in tau hyperphosphorylation in SPS-induced PTSD rats
Source: Aging (Albany NY). 2019 Sep 23;11(18):7978–95. doi: 10.18632/aging.102303 (PMC6782009; doi:10.18632/aging.102303)
Supplement: Supplementary Figures [file aging-11-102303-s001.pdf]

SUPPLEMENTARY FIGURES

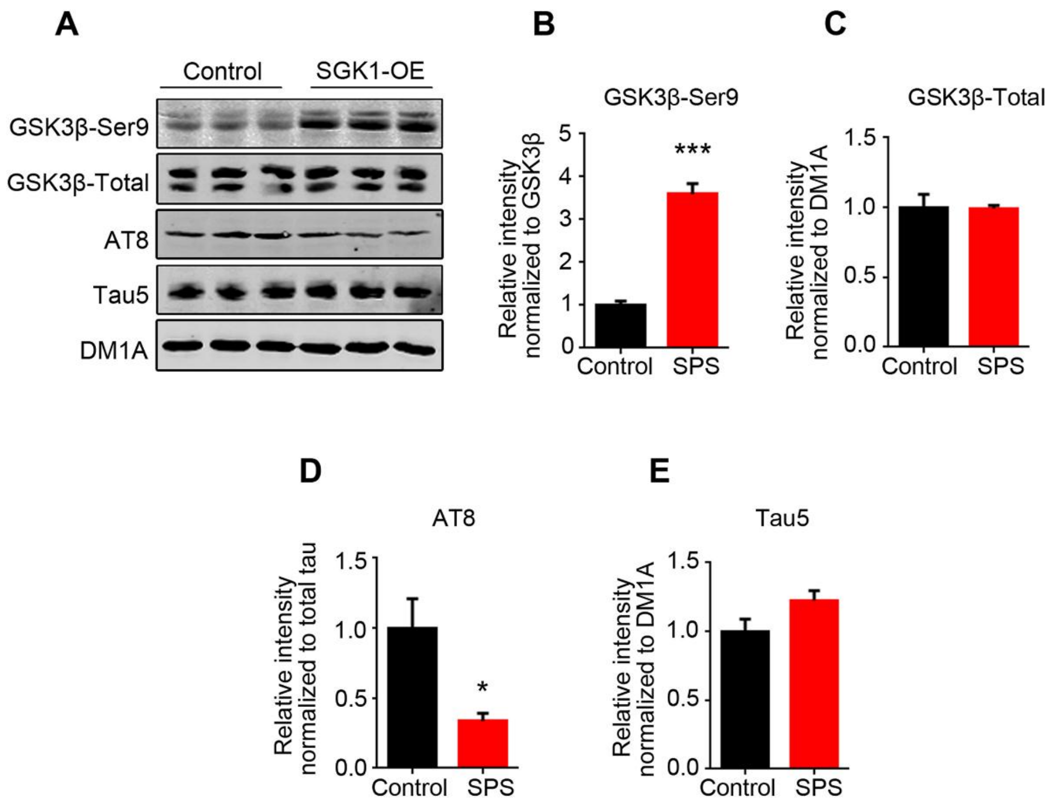

Supplementary Figure 1. Overexpression of SGK1 inhibits the GSK3β in HEK293/tau cells. HEK293/Tau cells were transfected with SGK1. (**A**) Western blot was performed to detect GSK3β and tau. SGK1 overexpression increased phosphorylation of GSK3β at Ser9 and decreased phosphorylation of tau at Ser202/Thr205 (AT8). (**B**–**E**) Quantitative analysis for blots in A.

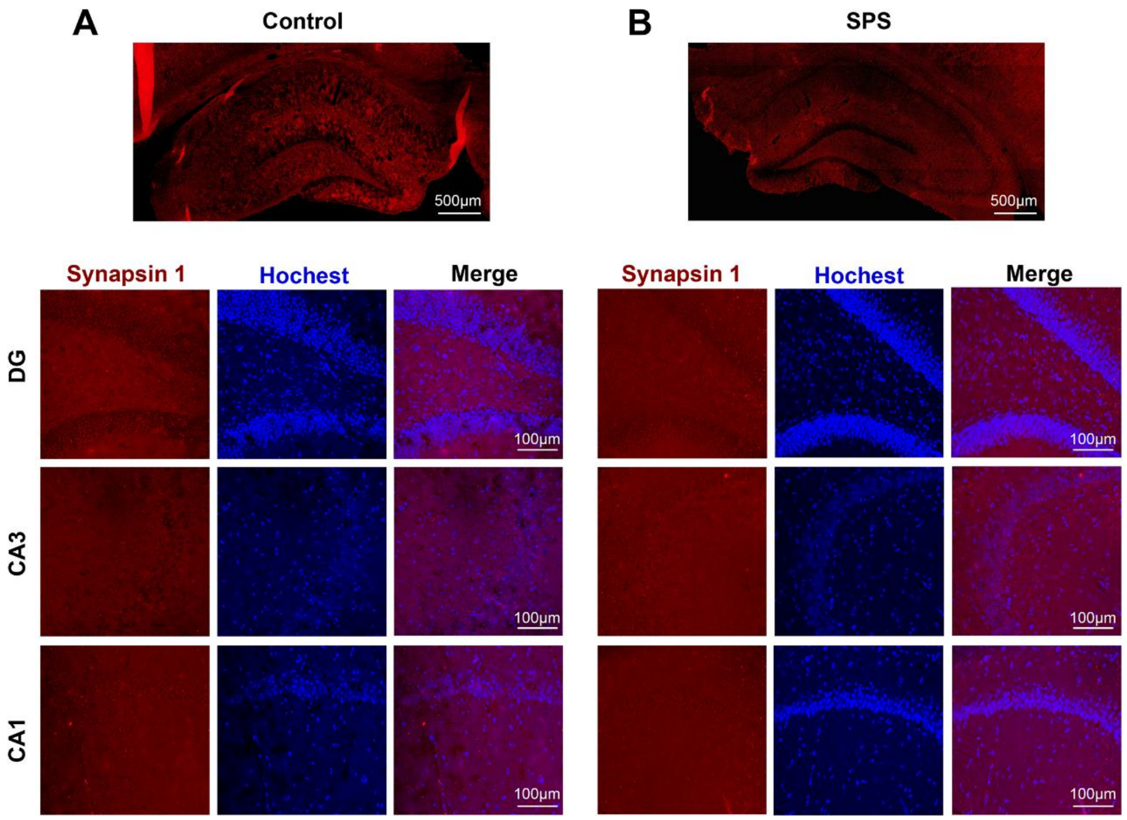

Supplementary Figure 2. Synapsin 1 in hippocampus of SPS rats. Immunofluorescence images stained with synapsin 1 (red) in hippocampus of control (**A**) and SPS rats (**B**). Nuclei were counterstained in hochest (blue). Upper row scale bar, 500µm; lower row scale bar, 100µm
